# Neighbor Overlap Is Enriched in the Yeast Interaction Network: Analysis and Implications

**DOI:** 10.1371/journal.pone.0039662

**Published:** 2012-06-26

**Authors:** Ariel Feiglin, John Moult, Byungkook Lee, Yanay Ofran, Ron Unger

**Affiliations:** 1 The Mina and Everard Goodman Faculty of Life Sciences, Bar-Ilan University, Ramat-Gan, Israel; 2 Institute for Bioscience and Biotechnology - University of Maryland, Rockville, Maryland, United States of America; 3 Laboratory of Molecular Biology, Center for Cancer Research, National Cancer Institute, National Institutes of Health, Bethesda, Maryland, United States of America; University of Rome, Italy

## Abstract

The yeast protein-protein interaction network has been shown to have distinct topological features such as a scale free degree distribution and a high level of clustering. Here we analyze an additional feature which is called Neighbor Overlap. This feature reflects the number of shared neighbors between a pair of proteins. We show that Neighbor Overlap is enriched in the yeast protein-protein interaction network compared with control networks carefully designed to match the characteristics of the yeast network in terms of degree distribution and clustering coefficient. Our analysis also reveals that pairs of proteins with high Neighbor Overlap have higher sequence similarity, more similar GO annotation**s** and stronger genetic interactions than pairs with low ones. Finally, we demonstrate that pairs of proteins with redundant functions tend to have high Neighbor Overlap. We suggest that a combination of three mechanisms is the basis for this feature: The abundance of protein complexes, selection for backup of function, and the need to allow functional variation.

## Introduction

The yeast Saccharomyces cerevisiae protein interaction network is probably the most studied protein interaction network both experimentally and computationally. The network has been shown to be scale free [1] i.e. the distribution of the degrees of the nodes follows a power law. In addition the network was shown to have large clustering coefficients (CC), [2], [3] meaning that neighbors of nodes in the network tend to interact amongst themselves (a property sometimes referred to as locality or modularity).

Here we explore a measure called Neighbor Overlap (NO) which reflects the number of common neighbors a protein pair has in the protein interaction network, normalized in various ways. Similar measures were used in previous studies to improve protein annotation, as it was expected that pairs with high NO should have similar function. Ravasz et al. utilized this measure to study the hierarchical organization of modularity in metabolic networks [4]. A related measure that calculates an edge clustering coefficient between directly connected nodes was used [5], [6] to detect communities in complex networks, including the *C. elegans* metabolic network.

In this work our aim is different. We study NO as a network property and show that it is highly enriched in the yeast protein interaction network compared to carefully designed control networks. Thus, we demonstrate that NO is an independent property of the yeast interaction network. Later we also explore the functional consequences of this observation.

The systematic analysis of large scale genetic and interaction data has led to intriguing observations regarding the ability of living organisms to sustain damage to their genes and still function effectively. It was demonstrated [7] that about 82% of the yeast proteins are non-essential in the sense that a single knockout of any of these genes leaves the organism viable, although about 15% show slower growth rate under rich medium conditions. While the lethality effect of genes is not easy to describe in such simple terms, it is reasonable that this kind of study can provide insight into robustness of biological systems. To further study the mechanisms used by biological systems to confer robustness, large scale experiments of double knockouts were performed. In these experiments pairs of genes are knocked out (or knocked down by RNAi). Two genes are said to participate in a genetic interaction if the effect of the double knockout is different from the expected effect of the combination of the two single knockouts. For example, a sample of 132 single knockouts in yeast for which all other second knockouts were performed [8] demonstrated that on average, each tested yeast gene was involved in a few dozen such genetic interactions.

In other studies the effect of double knockouts was tested on 424 genes involved in endoplasmic reticulum function [9] and on 743 genes related to DNA damage and transcription [10]. In these studies the phenotypic effect was measured on a continuous scale (i.e. not as a binary value of either synthetically lethal/sick or neutral) showing that many such genetic interactions have some, although small, effect. These experiments have also shown that some double mutants have an alleviating effect (i.e. the effect of the double knockout is smaller than the expected combination of the effect of the two single knockouts).

From these studies it became clear that backup patterns in living organisms are complicated. While in man-made systems, backup is often provided by simple pairing of parts that can directly substitute each other (e.g. a pair of pumps), the pattern revealed by the network of genetic interactions is much more complex.

Several studies have tried to link robustness of yeast against knockouts and mutations, to the structure of its protein interaction network. For example, the scale free characteristic of the yeast protein interaction network [1] has been associated with robustness to random mutations and vulnerability against direct attacks on the central hubs [11]. Additionally, using data from systematic single gene knockdown experiments, it was shown that hub genes tend to be more essential than genes with low connectivity [1] although the reason for this tendency is debated [12], [13].

When analyzing double knockdown experiments, Kelly and Ideker [14] emphasized the importance of genetic interactions that take place between proteins that reside in different modules, as they found that there are significantly more (in a ratio of about 1∶3.5) genetic interactions between pairs of proteins that are in different modules than between pairs of proteins that are in the same module. Their work was further extended by Ulitsky and Shamir [15] who found 140 cases of genetic interactions between modules.

When two proteins reside in different modules it is unlikely that they will share many neighbors. Thus, NO, which is the focus of our study, is a property of interactions that occur within a module. We show that high NO is associated with functional similarity and is enriched in pairs of proteins that participate in genetic interactions and that supply backup to each other. In the discussion we describe a few examples that demonstrate that high NO can stem from protein complexes, protein backup and functional variation and we argue that in many cases these factors are combined. Thus, this very simple measure correlates with significant factors that shape the protein-protein and genetic interaction networks.

When we want to show that any property of a complex network is either over or under represented compared to the expected value, a critical question is how to calculate the expected value. Almost always, it is impossible to derive analytical values for network properties. Thus, it is a common practice to create many randomized versions of the network, and consider the average frequency of the property in the randomized network as the expected value. This raises the question of how the randomization is done. In general, the randomization should be done in a way that will preserve as many of the other properties of the network, such that it will be clear that the claimed enrichment stands independently and is not a by-product of other properties. For example, in our case we want to show that the yeast protein interaction network is enriched with pairs of high NO. As we mentioned above, it was shown that the yeast protein interaction network is scale free and has high clustering coefficients. Thus, it is possible that the large number of pairs with high NO is a side effect of these properties and that every network that has these two features will have large number of pairs with high NO. To show that the yeast protein interaction network is specifically enriched with high NO we must therefore show that the overlap in the yeast network is higher compared to randomized networks that have similar scale free and cluster coefficient properties. Since this issue was the subject of several heated discussions [16], [17], [18], in this study we tried to be careful about the design of proper controls.

## Results

### Definitions of Neighbor Overlap

**Figure 1:**
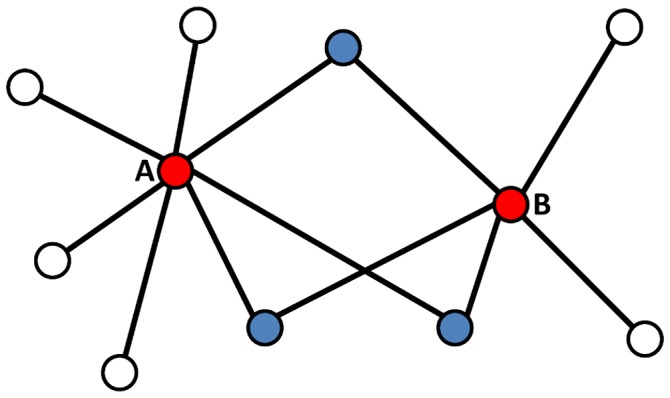
Schematic view of Neighbor Overlap. In the depicted example nodes A (degree = 7) and B (degree = 5) have 3 common neighbors. According to the definitions in the text, NOcount  = 3, NOnorm = 3/5 and NOjaccard  = 1/3.

NO is a measure of how many common neighbors a pair of proteins has in the protein interaction network. In our analysis, we use three forms of this measure. First we normalize the number of common neighbors to the minimum degree of the protein pair (NOnorm):
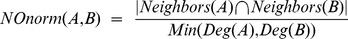



Second we use the Jaccard index (NOjaccard):
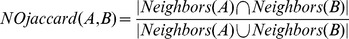



And third we use a simple count of common neighbors (NOcount):
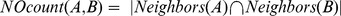



For example, in Figure 1 NOnorm = 3/5, NOjaccard = 3/9 = 1/3 and NOcount = 3. We note that this definition applies whether proteins A and B have a direct link or not.

### The Yeast Network is Enriched with High Neighbor Overlap

First we demonstrate that the yeast protein interaction network is enriched with protein pairs that have a high Neighbor Overlap, compared with 1000 control networks. These control networks were designed to preserve the degrees of each node in the original protein interaction network. Moreover, since protein interaction networks were shown to have modular characteristic [3], we further engineered the control networks to preserve the average cluster coefficient and a similar cluster coefficient distribution (Figure S1).

**Figure 2:**
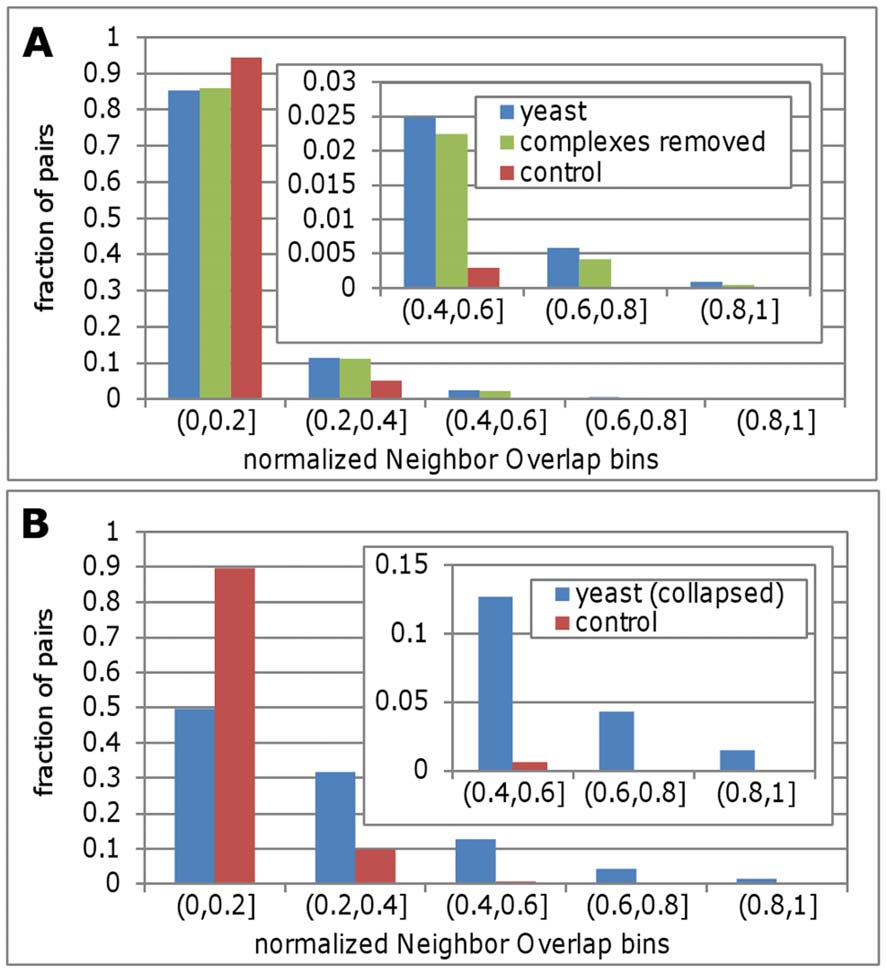
Enrichment of Neighbor Overlap in the yeast protein-protein interaction network – with and without complexes. Panel A shows the distribution of Neighbor Overlap using the NOnorm measure, for yeast (blue bars) versus control (red bars). Assessing the contribution of protein complexes to Neighbor Overlap was implemented by removing protein pairs that belong to the same complex from the original analysis (green bars). Panel B shows the yeast (blue bars) and control (red bars) NOnorm distributions on a collapsed version of the yeast interaction network. This was achieved by collapsing all proteins that are part of the same complex to a unified node and computing NOnorm values for the new network. To overcome difference in scale, the higher NOnorm bins are presented in the enlarged inserts. The figure shows that complexes contribute considerably to the NO enrichment, but even when complexes are removed the NO signal is strongly evident.

Figure 2 shows the NOnorm distribution in the yeast and control networks over five bins of increasing NOnorm values. These results demonstrate that the yeast protein interaction network is enriched with protein pairs for bins of NOnorm >0.2 (Figure 2A). The statistical significance of this result was verified by comparing the yeast and control distributions using the Mann Whitney U test (p<0.0001). Similar results are observed for the NOjaccard and NOcount measures (Figures S2 and S3, panel A).

To further neutralize the concern that the high modularity of the yeast protein interaction network is the main source of high NO, we checked the correlation between these attributes. Figure S5 reveals a low correlation (Pearson correlation coefficient = 0.17, p<0.0001) for NOnorm values versus the average cluster coefficient values for each pair. Although significant, the low correlation between these attributes indicates that the modular characteristic of the yeast protein interaction network can’t solely explain the high NO values. As evident in this plot, a wide spread of NO values is observed for any given cluster coefficient value. Taken together with the fact that our control networks preserve the cluster coefficient characteristics of the original yeast network, we conclude that Neighbor Overlap is an independent property of the yeast interaction network.

### Only Part of the High Neighbor Overlap Enrichment Originates in Protein Complexes

Two proteins that are part of the same protein complex are both likely to interact with other proteins that are part of the same complex. Therefore it is logical to assume that the abundance of protein complexes in yeast is a major source of high Neighbor Overlap. To assess the contribution of such protein pairs to the high NO enrichment, we removed all pairs reported to be in the same complex together. Our analysis is based on three datasets created by Pu et al. [19], Krogan et al. [20] and Gavin et al. [21].

The yeast NOnorm distribution after removing all protein pairs that were reported by Pu et al. (CYC2008 dataset) to be in the same complex is shown in the green bars of Figure 2A. High Neighbor overlap pairs are still over-represented in the yeast network when compared with the control network for bins of NOnorm>0.2 (Figure 2A). Although this over-representation is weaker than before, (the green bars are lower than the blue bars for the three highest bins of Figure 2A) the “complex removed” distribution is still significantly different from the control network based on the Mann Whitney U test (with p<0.0001). We performed the same analysis removing complexes that were reported by Krogan et al. and Gavin et al. and got similar results (Figure S6). Comparable results were also achieved for the NOjaccard and NOcount measures (Figure S2 and S3, panel A).

To further validate that protein complexes were not the only source of the high NO we created an additional control network. In this network we collapsed all proteins that were listed as being part of the same protein complex (in the CYS2008 dataset), to a single node. For example, if proteins A and B form a complex and either or both interact with C, we collapse A and B into a single node that interacts with C (see Methods). Here too, we created a set of 1000 control networks preserving both the degree and cluster coefficient characteristics of the network and re-performed the analysis. Figure 2B shows that the high NO enrichment persists under the conditions of this control as well. The distribution of the yeast and the control networks are significantly different using the Mann Whitney U test (with p<0.0001). This analysis was done for the NOcount and NOjaccard measures as well, and the results were similar (Figures S2 and S3, panel B). Note that counter intuitively, the NO values of the collapsed networks can be higher than in the original network that contains complexes. The fact that only pairs with non-zero NO values are considered and that all interactions of non collapsed nodes are assigned to the single collapsed node, contribute to this effect. Figure S4 demonstrates this effect in a “toy” example of a common scenario in a protein interaction network in which the nodes are highly connected within a complex but sparsely connected between complexes. Because of this effect it is not meaningful to compare the results of the original and collapsed networks but rather to compare each result with its corresponding control.

### High Neighbor Overlap Pairs have Higher Sequence Similarity than Low Ones

**Figure 3:**
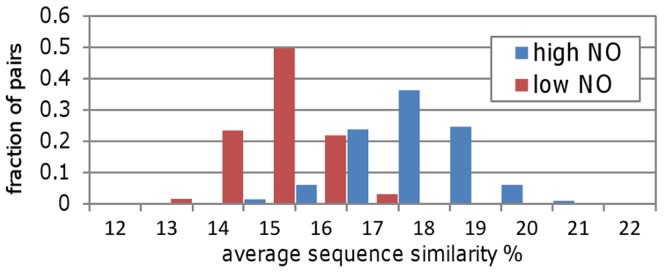
High Neighbor Overlap pairs have higher sequence similarity than low ones. The distribution of average sequence similarity for 1000 subsets (each of size 100) from the high (blue bars) and low (red bars) Neighbor Overlap groups are shown. These distributions indicate that high Neighbor Overlap pairs tend to have higher sequence similarity than low ones.

To start probing the relationship between pairs of proteins that share a high number of neighbors we checked if high NO protein pairs have higher sequence similarity than low ones. To this end we divided our data into two groups of high (NOnorm>0.5, n = 4,233) and low (0.5≥NOnorm>0, n = 294,307) NOnorm values and checked the sequence similarity levels in each group. To overcome the dramatic difference in size between the high and low sets, and in order to achieve a comparison that takes the degree of protein pairs into account, we used a sampling technique. We sampled 1000 subsets of the same size (n = 100) from the high and low sets, such that each pair in the high subset had a respective pair in the low subset with the same degree (for each of the two proteins). We calculated the average similarity for each subset in the high and low sets and compared their distribution. The results shown in Figure 3 clearly indicate that on average, high NO pairs have higher sequence similarities than low ones (p<0.0001 using the Mann Whitney U test to compare the distributions).

### Similar GO Annotations for High Neighbor Overlap Protein Pairs

**Figure 4:**
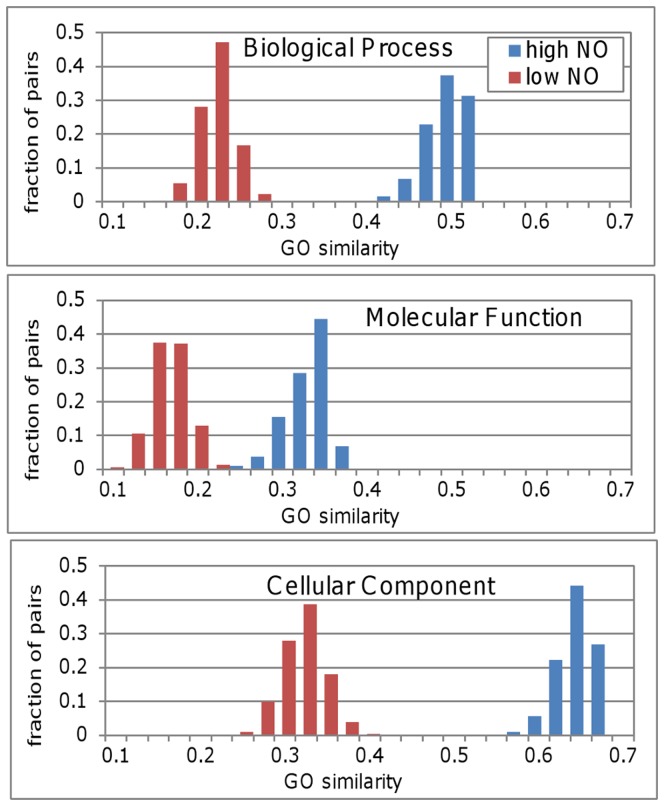
GO annotation similarity for high and low Neighbor Overlap groups. The distributions in each panel represent the GO annotation similarity of 1000 subsets each of size 100) from the high (blue bars) and low (red bars) Neighbor Overlap groups. The distributions for the three ontologies: Biological Process (top), Molecular Function (middle) and Cellular Component (bottom) show a marked separation between their GO similarities for pairs with high and low NO values.

To elucidate the functional ramification of high NO we checked if two proteins with high NO tend to have similar GO annotations. Using the sampling procedure described above we compared the GO similarity of high and low NO pairs for the three GO ontologies: Biological Process, Molecular Function and Cellular Component. The level of similarity was determined using the GOSim software package [22]. GOSim allows calculating the functional similarity of genes based on various normalization techniques for the GO terms of each protein. Figure 4 clearly shows that high NO protein pairs have a higher level of similarity for all three GO ontologies (p<0.0001 for all three ontologies using the Mann Whitney U test to compare the distributions).

### High Neighbor Overlap Pairs have Stronger Genetic Interactions than Low Ones

Quantitative measurements of genetic interactions can formally be defined by ε = W_ab_–W_a_×W_b_ [23] where W_a_ and W_b_ represent the fitness of organisms with either mutations a or b respectively and W_ab_ represents the fitness of organisms with both mutations a and b. The fitness of the mutated organisms is defined by their growth rates relative to that of wild-type organisms. Thus the ε value is expected to be close to zero for non-interacting gene pairs, less than zero for synthetic lethal (SL) and synthetic sick (SS) gene pairs and greater than zero for alleviating gene pairs. Many discussions have been devoted to understanding the functional meaning of SL and SS pairs (see for example Kupiec et al. [24]), however less emphasis has been given so far to the functional meaning of alleviating gene pairs. Nevertheless, it is reasonable to assume that protein pairs with either large negative or large positive ε values are functionally related.

**Figure 5:**
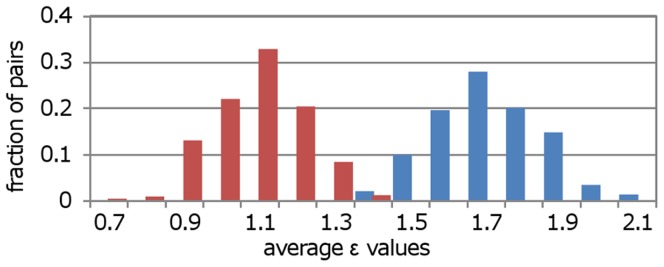
High Neighbor Overlap pairs have stronger genetic interactions than low ones. The distributions represent the average ε values of 1000 subsets (each of size 100) from the high (blue bars) and low (red bars) Neighbor Overlap groups. Clearly, the ε values are higher for the high than for the low group.

To demonstrate the relationship between Neighbor Overlap and genetic interactions we used a dataset created by Collins et al. [10], consisting of quantitative pair-wise genetic interaction measurements between 743 yeast genes involved in DNA damage and transcription. With the sampling procedure described above we compared the genetic interaction strength (i.e. absolute ε values) for the high and low NO groups. Figure 5 shows that high NO pairs have stronger genetic interactions than low ones (p<0.0001 using the Mann Whitney U test). Since genetic interactions are associated with backup of function between two genes we can suggest that high NO is indicative for gene backup.

### Redundant Gene Pairs are Enriched with High Neighbor Overlap

To further show that high NO indicates protein pairs with backup potential, we examined the NO values of redundant gene pairs. We expect that redundant gene pairs will have higher than average NO values. Thus, we analyzed two sets of gene pairs in which the two genes are mutually redundant; the first is a literature curated set and the second is computationally predicted set [25] (see methods for details). Crossing these datasets with our protein interaction network leaves us with 73 and 162 gene pairs respectively.

We begin by examining the fraction of pairs that have at least one common neighbor (non-zero NO) in the redundant sets and compared them with 1000 control sets. The control sets were designed so that every protein pair in the control had a respective pair with the same degree as in the redundant set being analyzed. Table 1 shows that the fraction of non-zero NO pairs is dramatically higher for the two redundant sets than for the control average (p<0.001 for both datasets in resampling tests).

**Table 1:** Non-zero Neighbor Overlap in redundant gene pairs.

Redundant gene sets	Neighbor Overlap>0	
	dataset	control
Literature Curated (n = 73)	68%	12% (±3)
Computationally Predicted (n = 162)	77%	13% (±2)

Next, we analyze the average NO values (NOnorm, NOjaccard and NOcount) only for pairs that have at least one common neighbor (NO>0) and compare them with 1000 control sets. The control sets in this case were designed to include only non-zero NO pairs with the same degree as the redundant set being analyzed. The results presented in Table 2 show that for all three measures NO values are significantly higher for the two redundant sets than for the control average (p<0.001 for all cases in resampling tests), strengthening the association of high NO with backup.

**Table 2:** Neighbor Overlap in redundant gene pairs for Non-zero Neighbor Overlap pairs.

Redundant gene sets(non-zero NO pairs only)	NOnorm		NOjaccard		NOcount	
	dataset	control	dataset	control	dataset	control
Literature Curated (n = 50)	0.49	0.30 (±0.01)	0.16	0.08 (±0.01)	5.2	2.1 (±0.2)
Computationally Predicted (n = 125)	0.34	0.18 (±0.01)	0.12	0.06 (±0.01)	4.5	2.4(±0.3)

## Discussion

Several previous studies have used high NO values for annotation. Samanta and Liang [26] used pairs of high NO to predict the function of one member of the pair whose function is unknown from the function of the other member. In Sun et al. [27] this measure was used as part of the inputs to a learning procedure whose goal was to assign function. Lin et al. [28] suggested that the small-world property (i.e. small diameter and a large clustering coefficient) implies high NO values and then went on to use this property to predict function. Other studies [5], [6] defined an edge clustering coefficient which was used to identify communities for various complex networks (mainly in social networks and in the *C. Elegans* metabolic network). Although this measure is similar to the NO measure, it has not been used to gain biological insight as to the origin and functional implications of this property. Additionally, the edge clustering coefficient is only defined for pairs of nodes that have a direct link. In our study, NO values were calculated for 298,540 pairs out of which only 10,828 pairs (about 4%) have a direct link; therefore the edge clustering coefficient is not applicable to NO analysis.

Several special features such as scale free topology and modular organization have been shown for the yeast protein interaction network and much effort has been invested in understanding the functional significance of these characteristics. Neighbor Overlap is an additional interesting characteristic which may have important functional implications. We have shown that the yeast protein interaction network is enriched with protein pairs that have high Neighbor Overlap compared with control networks that preserve degree and clustering coefficient characteristics. These two characteristics are intrinsic topological parameters of the network. However, we did not control for additional biological parameters like sequence similarity and GO similarity since, as we show, they are inherently related to the NO property. The sequence similarity and the similar GO annotations suggest that high NO pairs tend to have similar functions. The association with genetic interactions and enrichment of redundant genes with high NO pairs indicate that these functionally similar high NO pairs may be part of an effective backup mechanism that contributes to the robustness of the organism.

We suggest that the enrichment of Neighbor Overlap in the yeast protein interaction network is associated with at least three different, but related, mechanisms. One is from the existence of complexes, the second is associated with functional backup and the third is to allow functional variation.

We show three examples, one for each mechanism, and briefly describe the interactions with their common neighbors.

The definition of what constitutes a complex varies and as a result the estimation of the number of complexes in cells varies significantly [19], [20], [21]. Nevertheless, it is clear that protein complexes are abundant. When two proteins are part of the same complex, it is clear that their interaction pattern will be similar. While some variation may occur due to geometrical or temporal considerations, it is likely that proteins within a complex will interact with the same set of proteins. Indeed, our data (Figures 2 and S6) show that a considerable portion of the enrichment in Neighbor Overlap comes from this attribute. One example is the pair of proteins Vph1 and Stv1 which are isoforms of the subunit “a” of Vascular ATPase (V-ATPase) V_0_ domain. V-ATPases are ATP-dependent proton pumps that acidify intracellular vacuolar compartments [29]. In our network, Vph1 and Stv1 have 13 and 15 neighbors respectively, of which 9 are common: Vma2, Vma4 - 8, Vma10, Vma13 and Tpf1 (NOnorm = 0.69, NOjaccard = 0.47 and Nocount = 9). Their common interaction partners are all other subunits of the V-ATPase complex and are the source of the high NO in this case.

Another reason for the enrichment of Neighbor Overlap may be related to selection for functional backup. In these cases, the two proteins that share neighbors can substitute each other. One such example in our data is Mkk1 and Mkk2 which are mitogen activated protein kinases (MAPKs), involved in the cell wall integrity pathway [30], [31]. In our network, Mkk1 and Mkk2 have 11 and 41 neighbors respectively, of which 5 are common: Bck1, Slt2, Spa2,Sph1 and Atp2 (NOnorm = 0.45 and NOjaccard = 0.11 and NOcount = 5). Two of which are other kinases immediately upstream (Bck1) and downstream (Slt2) in the signaling pathway. Spa2 and Sph1 are scaffolding proteins [32], [33]. It was shown that the signal transduction pathway is fully functional with either one of these two proteins [34], [35]. Thus, the high NO in this case is a result of the similar neighborhoods required for two proteins to carry out the same (or a very similar) task.

Another possible reason for the enrichment of high NO pairs is that an organism may have proteins with the same basic function in many different contexts and nuances. An example of this is illustrated by Dig1 and Dig2 which are two regulatory proteins from the MAPK signaling cascade [36]. In our network Dig1 and Dig2 have 12 and 14 neighbors respectively of which 6 are common: Fus3, Kss1, Ste12, Cln1, cln2 and Srp1 (NOnorm = 0.5 and NOjaccard = 0.3 and NOcount = 6). Ste12 activates signal-responsive transcription required for pheromone response in haploid yeasts and filamentous growth as a result of limiting nutrients [37]. Regulation of Ste12 appears to involve the two MAP kinases Fus3 and Kss1, which phosphorylate Ste12, Dig1 and Dig2, which in turn inhibit the Ste12 function [38]. Additionally, Fus3 and Kss1 take part in the control over G1 arrest by repressing transcription of G1/S cyclin genes Cln1, Cln2 and Clb5 [39]. Since Ste12 is involved in separate signal paths that result in unique behavior, its activity must be tightly regulated. Dig1 and Dig2 are both negative regulators of Ste12 in both the pheromone and the filamentous growth response [40]. Dig1 and Dig2 have been shown to be functionally redundant; that is the individual disruption of either one has no apparent phenotype while their simultaneous disruption results in extensive filaments and elevated expressions of pheromone responsive genes [36], [40], [41]. Despite this apparent redundancy, Dig1 and Dig2 inhibit Ste12 through independent mechanisms. It has been shown [42] that while Dig1 binds to a central region of Ste12 (residues 309 to 547), Dig2 binds to its DNA binding domain (residues 1 to 215). A possible hypothesis accounting for these separate interaction sites is that Dig2 directly modulates the capability of Ste12 to bind to the pheromone response element by blocking its DNA binding site. In contrast, Dig1 may interact not by modulating the DNA binding but rather by interacting with the DNA bound Ste12 and preventing its transcriptional activation. The origin of high NO in this example is from five proteins all involved in this regulatory pathway. Thus, this is an example of functionally, rather than mechanistically, redundant proteins and it underlines an important feature necessary for the fine tuning of regulatory pathways.

Although we have suggested three separate mechanisms as the source of the high NO in yeast, it is clear that these mechanisms are intertwined. High NO pairs that are in complexes are likely to be relevant for backup and/or variation as well. For example, although Vph1 and Stv1 have high NO partly as a consequence of being members of the same complex, they have also been shown to have a partially compensatory relationship. Moreover, it was suggested that they have distinct variant roles in targeting the V-ATPase complex to different cellular compartments [29]. Similarly, as we have discussed, Mkk1 and Mkk2 are known to provide backup but they too are suspected to have different regulatory roles in promoting cell wall integrity [43].

We must keep in mind that many of the gene pairs that provide the combination of back-up and functional variation may come from gene duplication: Immediately after the duplication, the function of the two genes and their product would have been identical and the pair must have served mainly for backup function. However, with time, only pairs that offer significant functional variation [44] or regulatory control variation [45] may have survived. It is therefore a combination of these mechanisms in yeast that is the major source of the high NO. This hypothesis is supported by the observation that pairs with high NO tend to have higher sequence similarity and by the fact that the redundant gene sets for which we showed high NO, are based [25] on duplicated yeast genes. The number of duplicated gene pairs is presumably higher in the yeast Saccharomyces cerevisiae because of its ancient whole genome duplication [44]. This is relevant to our discussion since it has been suggested [46] that paralogs resulting from the whole-genome duplication are more likely to share interaction partners and biological functions than smaller-scale duplicates. On the other hand, it has been demonstrated [47] that the age of the duplication has a major effect on function diversification of the proteins, although interestingly even after duplication, proteins tend to maintain their domain architecture. The differences between whole genome duplications and more local duplications leave open for further studies the question of whether NO will be lower in organisms that did not undergo massive duplication.

In summary, we have shown that NO, although simple and straightforward, is an informative property of the yeast protein interaction network that reflects the complicated relationship between proteins. Clearly, the fact that a pair of proteins has a high NO does not always have obvious functional implications; but having similar neighborhoods is often a consequence of the intricate functional relationship between proteins.

## Methods

### Yeast Protein Interaction Network Data

The protein interaction network was downloaded from the DIP database (using the version published on the 27/10/11, filename: Scere20111027.txt) and comprises 5,009 genes and 21,894 reciprocal interactions (43,788 non-reciprocal ones). Each gene has an average of 8.74 interacting partners (degree), and the degree distribution has a scale free topology (linear distribution on a log-log scale). The network is predominantly one giant connected component of 4,958 genes with an additional 24 isolated pairs and one isolated triplet. The evidence for these interactions is based mainly on yeast Two-Hybrid assay and Affinity Purification followed by Mass Spectrometry. For the analysis presented in this paper we considered only protein pairs in which both proteins have at least 5 interacting partners resulting in 298,540 pairs with non-zero NO. A list of all pairs with NOnorm>0.5 (4,233 pairs) is given in Table S1. The average cluster coefficient calculated for this network is 0.322.

### Control Networks

Degree preserving networks were created by shuffling the original network. This was done by randomly choosing an existing pair of edges in the original yeast network and rewiring them. In this procedure, for each removed edge another edge is gained and thus the degree of each node is preserved, similar to the method described in [48]. For example, edges A-B and C-D were rewired to be A-C and B-D, provided they did not already exist. 1000 such control networks were created. However, a byproduct of shuffling the original yeast network is a reduction in the average cluster coefficient of the control networks. Therefore we implemented a shuffling algorithm that takes the control networks and reshuffles them such that only rewiring steps that increase the local average cluster coefficient are accepted. We continued this “biased” rewiring until the original average cluster coefficient was restored. We note that under the degree preserving constraint this procedure also preserves the cluster coefficient distribution to a large extent (Figure S1).

### Collapsed Network

To create a “complex free” protein interaction network we collapsed all proteins that were documented in [19] to be in the same biological complex. All proteins that were part of the same complex were collapsed and unified into a single node that interacts with all proteins that previously interacted with the proteins of the complex. If a protein was part of more than one complex it was collapsed to all. The new network comprised 3,637 nodes and 9,084 reciprocal interactions (18,168 non-reciprocal ones).

### Sequence Similarity

The similarity between protein sequences was determined using the global alignment algorithm “Needle” from the EMBOSS package with the default parameters [49].

### GO Analysis

The similarity between two genes was computed using the GOSim R package [22].A yeast database (org.Sc.sgd.db) was added to the package. We used the getGeneSim function with default parameters.

### Genetic Interactions

The genetic interaction ε values were downloaded from the supporting information of [10] (filename: Chromosome biology genetic interaction scores.xls). After removing genes that appeared more than once and crossing the data with the yeast network we were left with 676 genes. For our analysis we discarded interactions that were not symmetric (i.e. ε (A,B)≠ ε (B,A)). Dividing this data into high (NOnorm>0.5) and low (0.5≥NOnorm>0) groups left us with 201 and 9,935 pairs in each group respectively.

### Redundant Gene Sets

The two redundant gene sets [25] were created by the authors based on the following criteria: The literature curated set comprises 84 paralogous gene pairs that have documentation of functional overlap (from non high throughput studies) as well as experimental validation of a compensatory relationship. The computationally predicted set comprises 161 gene pairs that: [a] are paralogs based on BLASTP (E<10^−20^), [b] have a mean expression similarity <0.3 and [c] have at least 5 connections in the protein interaction network derived from the GRID database. Crossing these data sets with the interaction network left us with 73 pairs for the literature curated set and 162 pairs for the computationally predicted set.

## Supporting Information

Figure S1 **Cluster Coefficient distribution for the yeast and control networks.** Cluster Coefficient distribution across 10 bins for the yeast (blue bars) and the average of 1000 control networks (red bars).
(TIF)

Figure S2 **Enrichment of Neighbor Overlap in the yeast protein-protein interaction network using NOjaccard – with and without complexes.** Panel A shows the distribution of Neighbor Overlap using the NOjaccard measure, for yeast (blue bars) versus control (red bars). Assessing the contribution of protein complexes to Neighbor Overlap was implemented by removing protein pairs that belong to the same complex from the original analysis using three different complex lists created by Pu et al., Krogan et al. and Gavin et.al (green, purple and aqua bars respectively). Panel B shows the yeast (blue bars) and control (red bars) NOjaccard distributions on a collapsed version of the yeast interaction network. This was achieved by collapsing all proteins that are part of the same complex to a unified node and computing NOjaccard values for the new network. To overcome difference in scale, the higher bins are presented in the enlarged inserts. The figure shows that complexes contribute considerably to the NO enrichment, but even when complexes are removed the NO signal is strongly evident.
(TIF)

Figure S3 **Enrichment of Neighbor Overlap in the yeast protein-protein interaction network using NOcount – with and without complexes.** Same as figure S2 but using the NOcount measure.
(TIF)

Figure S4 **Original versus collapsed NO values.** Using a “toy” network, this figure demonstrates that in a typical scenario in which the nodes are highly connected within a complex but sparsely connected between complexes, the NO distribution is shifted to the right for the collapsed network. The original network (top left) and its NOnorm distribution (bottom left) are shown. When collapsing the network by unifying proteins from the same complex into a single node, the collapsed network (top right) has a NOnorm distribution with higher NO values (bottom right).
(TIF)

Figure S5 **Correlating Neighbor Overlap and average Cluster Coefficients.** A plot of NOnorm values versus the average cluster coefficient values for each pair is shown. While there is some correlation (Pearson correlation coefficient  = 0.17 which is statistically significant (p<0.0001)), it is clear that there is a wide spread of Neighbor Overlap values for any given cluster coefficient value. This observation supports our claim the contribution of the high clustering coefficient of the yeast network to the high NO values is limited.
(TIF)

Figure S6 **Enrichment of Neighbor Overlap in the yeast protein-protein interaction network using NOnorm – with and without complexes.** Panel A shows the distribution of Neighbor Overlap using the NOnorm measure, for yeast (blue bars) versus control (red bars). To Assess the contribution of protein complexes to Neighbor Overlap, protein pairs that belong to the same complex were removed from the original analysis using three different complex lists created by Pu et al., Krogan et al. and Gavin et al. (green, purple and aqua bars respectively, A). To overcome difference in scale, the higher NOnorm bins are presented in the enlarged inserts. All analyses show that complexes contribute considerably to the NO enrichment, but even when complexes are removed the NO signal is strong.
(TIF)

Table S1 **The table lists the details of protein pairs for which NOnorm >0.5 and the degree of both proteins in the protein interaction network is ≥5.**
(XLS)
