# Supplementary figures and images for: Neighbor Overlap Is Enriched in the Yeast Interaction Network: Analysis and Implications
Source: PLoS One. 2012 Jun 26;7(6):e39662. doi: 10.1371/journal.pone.0039662 (PMC3383679; doi:10.1371/journal.pone.0039662)

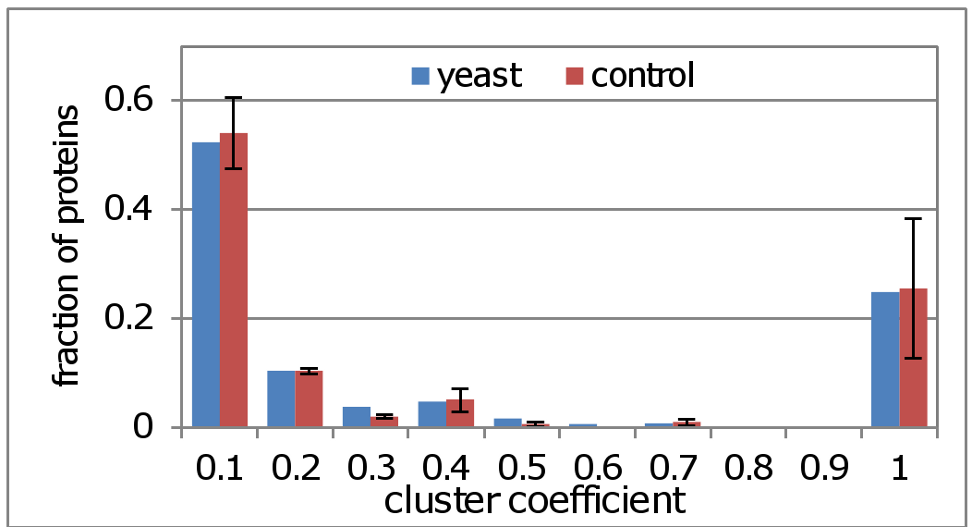

Supplement: Figure S1 — Cluster Coefficient distribution for the yeast and control networks. Cluster Coefficient distribution across 10 bins for the yeast (blue bars) and the average of 1000 control networks (red bars). (TIF) [file pone.0039662.s001.tif]

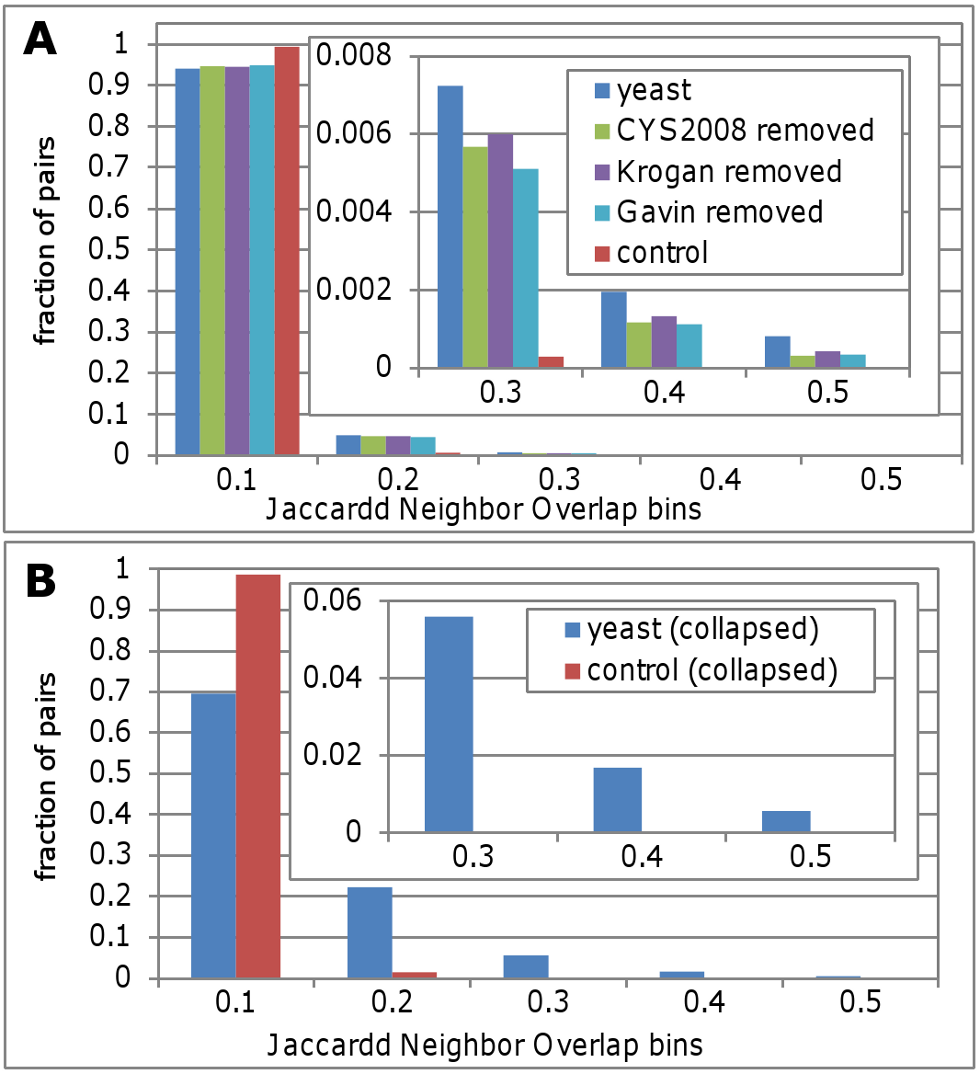

Supplement: Figure S2 — Enrichment of Neighbor Overlap in the yeast protein-protein interaction network using NOjaccard – with and without complexes. Panel A shows the distribution of Neighbor Overlap using the NOjaccard measure, for yeast (blue bars) versus control (red bars). Assessing the contribution of protein complexes to Neighbor Overlap was implemented by removing protein pairs that belong to the same complex from the original analysis using three different complex lists created by Pu et al., Krogan et al. and Gavin et.al (green, purple and aqua bars respectively). Panel B shows the yeast (blue bars) and control (red bars) NOjaccard distributions on a collapsed version of the yeast interaction network. This was achieved by collapsing all proteins that are part of the same complex to a unified node and computing NOjaccard values for the new network. To overcome difference in scale, the higher bins are presented in the enlarged inserts. The figure shows that complexes contribute considerably to the NO enrichment, but even when complexes are removed the NO signal is strongly evident. (TIF) [file pone.0039662.s002.tif]

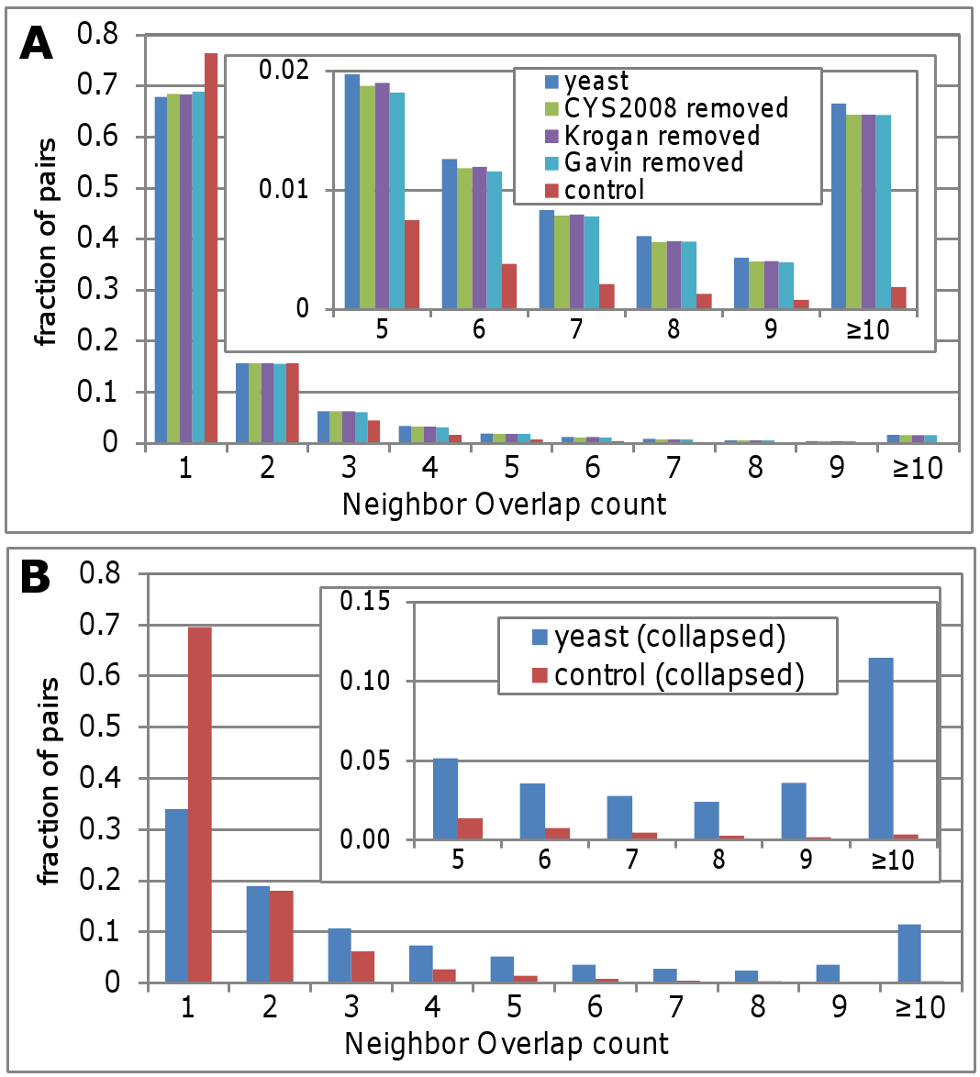

Supplement: Figure S3 — Enrichment of Neighbor Overlap in the yeast protein-protein interaction network using NOcount – with and without complexes. Same as figure S2 but using the NOcount measure. (TIF) [file pone.0039662.s003.tif]

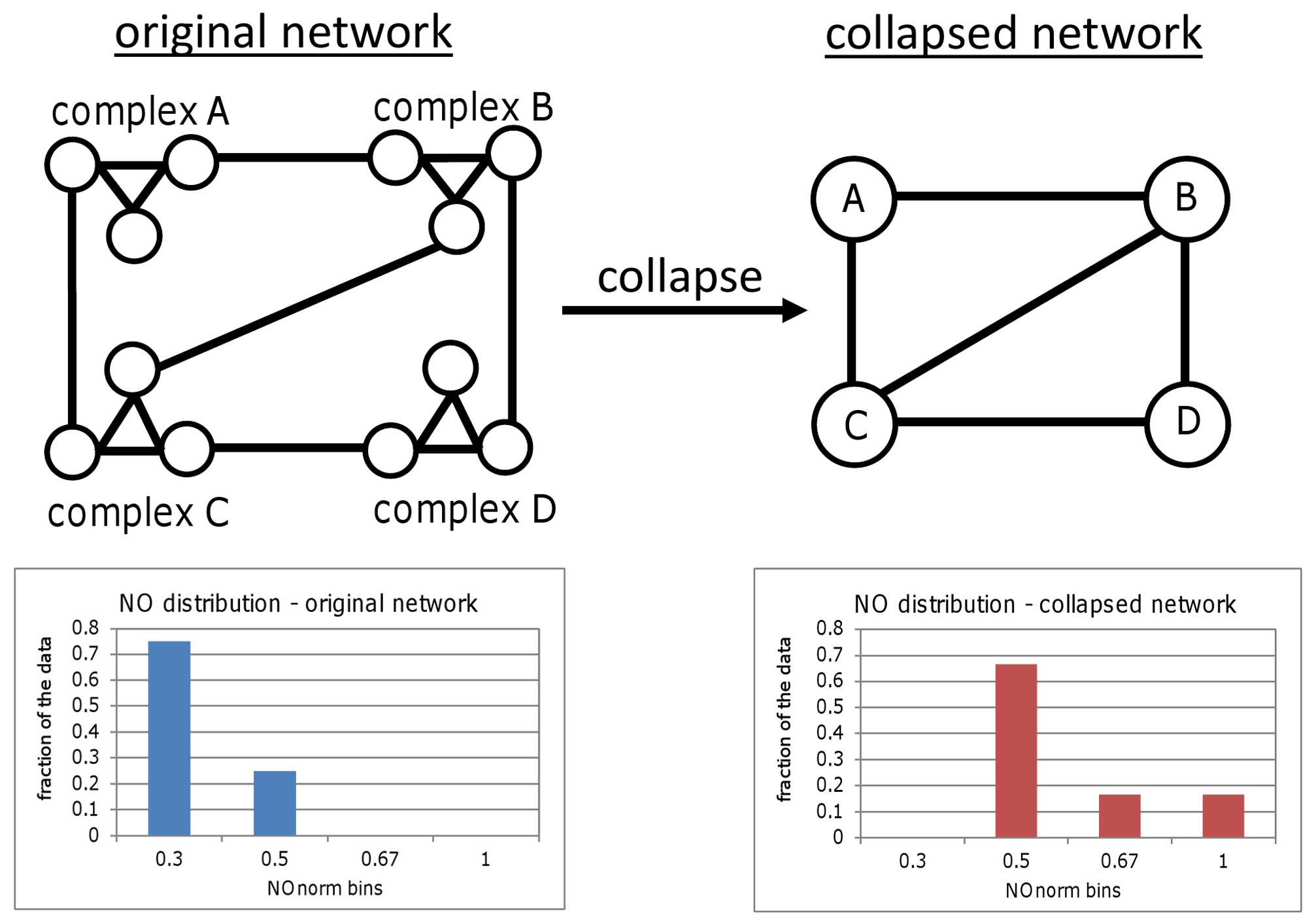

Supplement: Figure S4 — Original versus collapsed NO values. Using a “toy” network, this figure demonstrates that in a typical scenario in which the nodes are highly connected within a complex but sparsely connected between complexes, the NO distribution is shifted to the right for the collapsed network. The original network (top left) and its NOnorm distribution (bottom left) are shown. When collapsing the network by unifying proteins from the same complex into a single node, the collapsed network (top right) has a NOnorm distribution with higher NO values (bottom right). (TIF) [file pone.0039662.s004.tif]

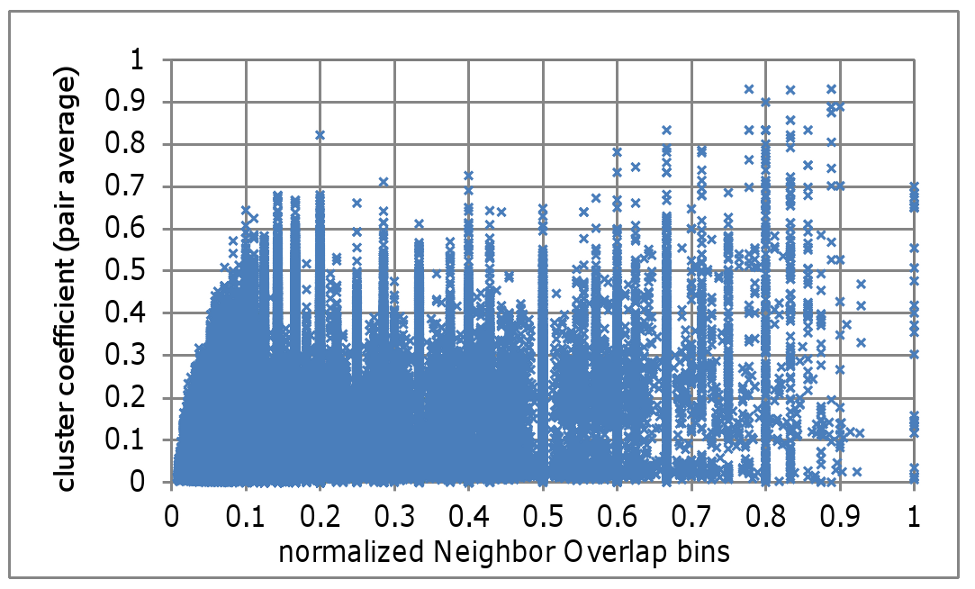

Supplement: Figure S5 — Correlating Neighbor Overlap and average Cluster Coefficients. A plot of NOnorm values versus the average cluster coefficient values for each pair is shown. While there is some correlation (Pearson correlation coefficient = 0.17 which is statistically significant (p<0.0001)), it is clear that there is a wide spread of Neighbor Overlap values for any given cluster coefficient value. This observation supports our claim the contribution of the high clustering coefficient of the yeast network to the high NO values is limited. (TIF) [file pone.0039662.s005.tif]

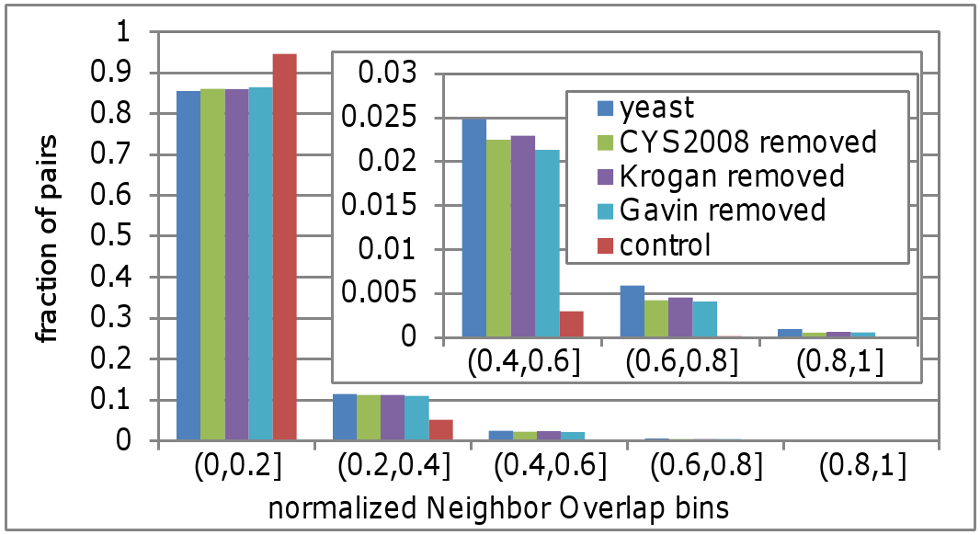

Supplement: Figure S6 — Enrichment of Neighbor Overlap in the yeast protein-protein interaction network using NOnorm – with and without complexes. Panel A shows the distribution of Neighbor Overlap using the NOnorm measure, for yeast (blue bars) versus control (red bars). To Assess the contribution of protein complexes to Neighbor Overlap, protein pairs that belong to the same complex were removed from the original analysis using three different complex lists created by Pu et al., Krogan et al. and Gavin et al. (green, purple and aqua bars respectively, A). To overcome difference in scale, the higher NOnorm bins are presented in the enlarged inserts. All analyses show that complexes contribute considerably to the NO enrichment, but even when complexes are removed the NO signal is strong. (TIF) [file pone.0039662.s006.tif]
